# Assessment of transcultural psychotherapy to treat resistant major depressive disorder in children and adolescents from migrant families: Protocol for a randomized controlled trial using mixed method and Bayesian approaches

**DOI:** 10.1002/mpr.1847

**Published:** 2020-09-12

**Authors:** Jonathan Lachal, Marie Rose Moro, Emilie Carretier, Amalini Simon, Caroline Barry, Bruno Falissard, Alexandra Rouquette

**Affiliations:** ^1^ AP‐HP Maison de Solenn Hôpital Cochin Paris France; ^2^ Université de Paris PCPP Boulogne‐Billancourt France; ^3^ CESP Fac. de Médecine ‐ Université Paris‐Sud Fac. de Médecine ‐ UVSQ INSERM DevPsy, Université Paris‐Saclay Villejuif France; ^4^ Public Health and Epidemiology Department AP‐HP Bicêtre Hospital Le Kremlin‐Bicêtre France

**Keywords:** child, depression, mixed method, transcultural, treatment

## Abstract

**Objectives:**

Cultural variations complicate psychiatric care, especially for migrant children. Transcultural psychotherapy (TCP) is an original psychotherapeutic technique developed to address complex situations of resistant mental disorders in the context of migration. This research will aim to assess the efficacy, the acceptability, and describe the therapeutic processes of TCP for the treatment of depression in first or second generation of migration children and adolescents.

**Method:**

Mixed method study using a multicenter, Bayesian randomized clinical trial with blinded evaluation of the primary outcome. Two parallel groups of 40 children or adolescents from 6 to 20 years old and their family will be included. In the experimental group, patients will attend six sessions of transcultural therapy in addition to usual care.

**Results:**

The improved Clinical Global Impression scale scores at 6 months will be compared across groups. Qualitative analysis of families and therapists' interviews will allow to specify the therapeutic processes and acceptability of the therapy.

**Conclusion:**

The findings will encourage the development and routinization of TCP for second‐line use and its adaption as a first‐line technique in this population.

**Abbreviations**
CDRS‐R: Children's Depression Rating Scale‐Revised
CGI: Clinical Global Impression
CNIL: Commission Nationale de l'Informatique et des Liberté (French and European General Data Protection Regulation)
iCGI: improved Clinical Global Impression
SD: standard deviation
STAI‐C: State‐Trait Anxiety Inventory for Children
TCP: transcultural psychotherapy

## 1 INTRODUCTION

Migrant children represent 9.4% (47.3 million) of the European population and come, for a large part, from low‐ and middle‐income countries. In France, one in three children born has at least one parent who comes from elsewhere and 17% of children under 18 are migrants (INSEE, 2016). This proportion continues to rise and as they may differ in physical appearance, language, religion, and culture from the nonmigrant population (Hernandez, 2010), psychiatric care of immigrated families constitutes a growing challenge in many Western societies (Ceri et al., 2017; Georgiades, Paksarian, Rudolph, & Merikangas, 2018).

In Europe, migrant children have been shown to exhibit a 5%–10% higher risk of internalizing problems in various studies (Belhadj Kouider, Koglin, & Petermann, 2014; Kirkcaldy, Furnham, & Siefen, 2011; Reijneveld, Harland, Brugman, Verhulst, & Verloove‐Vanhorick, 2005). First generation of migration children shows more symptoms of depression, anxiety, post‐traumatic stress, or other mental disorders linked to internalizing disorders (impairment in school functioning, sleep disturbance, or suicidal ideation) than nonmigrant children (Fazel, Reed, Panter‐Brick, & Stein, 2012; Gaber et al., 2013; Jaycox et al., 2002). In recent studies, lifetime prevalence of most mental disorders was around 35% higher among second‐generation migrant children than among natives (Ceri et al., 2017). They showed particularly high rates of depression, anxiety, and post‐traumatic stress disorder which may lay on particular psychological vulnerabilities in migrant children (Chau, Baumann, Kabuth, & Chau, 2012; Kwak, 2003; Smokowski & Bacallao, 2007; Stevens & Vollebergh, 2008).

Treatment of depressive disorders in migrant children is a real challenge. Indeed, they are known to exhibit specific symptoms which may hinder the diagnosis and that cultural variations affect symptoms and clinical presentation of the entire range of mental health problems (Baker, 2001; Choi, 2002; Nguyen, Huang, Arganza, & Liao, 2007). Access to mental health services is also known to be more difficult for migrant children, particularly when they present symptoms of internalizing disorders that are more difficult to identify for caregivers (Leong & Lau, 2001; Nadeau, Jaimes, Johnson‐Lafleur, & Rousseau, 2017). Moreover, being part of an ethnic minority is known as a main risk factor of treatment dropout in youth mental healthcare (de Haan, Boon, de Jong, & Vermeiren, 2018).

As shown by the numerous cultural adaptations of research and therapeutic settings done over the world, culture has always been a concern in psychiatry (Kirmayer & Minas, 2000). Many care settings are currently designated to treat migrant families in America, Europe, or Australia, mostly based on the host country's migration patterns and citizenship model. Transcultural psychotherapy (TCP) is an original psychotherapeutic technique developed to meet specific requirements of migrants suffering from mental health issues. Its theoretical and methodological foundations rest on Devereux's (1967) work in ethnopsychiatry, and Nathan (1986) developed a psychotherapeutic technique intended to first‐generation migrants. Then, Moro (1998) adapted this technique to second‐generation which is now fully formalized as a second‐line treatment after failure of standard care. It combines cultural mediation techniques with elements from psychoanalytic parent–infant therapy, narrative therapy, and systemic and psychoanalytic family therapy (Sturm, Heidenreich, & Moro, 2009). Currently commonly used in France and in various European and American countries, TCP has shown its value in different contexts—first‐ or second‐generation migrants, refugees, unaccompanied minors, and so on—in numerous qualitative studies and clinical case reports (Moro, 2003, 2014; Sturm, Nadig, & Moro, 2011). However, it has never been evaluated using a design providing a high level of evidence, such as a randomized controlled trial.

In practice, the standard psychotherapeutic care offered to children and adolescents with depression does not usually lead to complete remission in migrant patients (de Haan, Boon, de Jong, Hoeve, & Vermeiren, 2013). These patients often present complex clinical depression with numerous comorbidities and require long care, with a substantial increase in human and financial resources, resulting in longer states of depression, which often become chronic (de Haan et al., 2013). We hypothesize that, when remission is not complete after 6 months of standard care, TCP is more effective to treat depression in migrant children and more acceptable for migrant families than the continuation of the standard psychotherapeutic care.

In this study, we present the study protocol of the randomized controlled trial we planned to evaluate TCP in migrant children using mixed methods and Bayesian approaches. Quantitative data will allow us to evaluate the efficacy of TCP using Bayesian inference which allows accounting for both a prior knowledge on the parameters to be determined as well as observations actually made on patients during the study. Qualitative aspects will help to specify the therapeutic processes to enhance the reproducibility and transmissibility: What are the principal therapeutic aspects during the group session? How to make them more effective? Finally, acceptability data will allow us to define the limits of its indications.

### 1.1 Objectives

**FIGURE 1:**
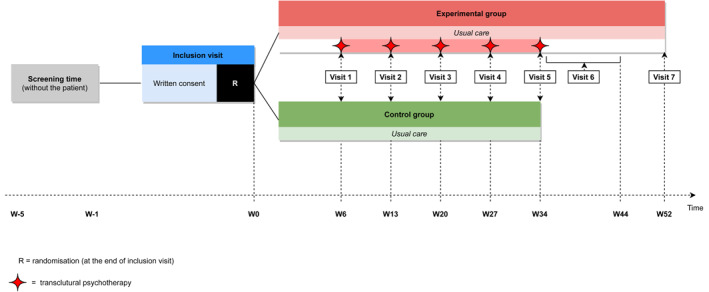
Chart of the conduct of the study

The main objective is to assess the efficacy of TCP to treat depression resistant to a first‐line therapy in first or second generation of migration children and adolescents, by comparing the percentage of patients in remission at 28 weeks after the start of TCP versus usual care (Figure 1). Secondary objectives consist in comparing the course of the severity of depression and of the level of depressive and anxiety symptoms in both groups over the first 34 weeks after inclusion, in evaluating the persistence of the efficacy of TCP at 1 year, and finally in describing the therapeutic processes that enabled the improvement of patients treated by TCP and the perceived efficacy and acceptability for the patient and the family.

## 2 METHOD

This is a multicenter, parallel group, Bayesian randomized clinical trial with blinded evaluation of the primary outcome and semi‐structured interviews for qualitative approach. The primary outcome—remission—will be assessed after the end of the follow‐up of the last included participant by three independent experts unaware of the received treatment who will watch the filmed semi‐structured clinical interview conducted by a psychologist at 34 weeks after inclusion (i.e., 28 weeks after the start of the TCP). The study was approved by the Research Ethics Board of Sud Mediterranean IV (CPP 19.10.01; January 13, 2020) and meet the requirements of the French and European General Data Protection Regulation (CNIL). The study is funded by a grant from Programme Hospitalier de Recherche Clinique—PHRC 2018 (French Ministry of Health)—and is registered on the website http://clinicaltrials.gov/ (NCT04206969).

### 2.1 Population and sampling

Participants are recruited in six outpatient hospitals and psychological care centers in France in which every main therapist providing TCP has followed the same training. The study involves first and second generation of migrant children and adolescents suffering from depression resistant to a first‐line therapy, their families, and the therapists. The recruitment is planned to be from January 2020 to July 2022.

#### 2.1.1 The child or adolescent and his/her family

Children and adolescents (the participants) referred to the transcultural consultation of one of the recruiting centers because they suffer from a depressive disorder resistant to standard management are eligible to be included in the study. TCP involves parents and siblings, so they are also present and active at each endpoint measure and involved in the semi‐structured interviews on acceptability of the therapy.

As mentioned above, the diagnosis of depressive disorders in migrant children and adolescent may be hindered by the effects of the culture (Haroz et al., 2017). To the well‐known important variability of depressive symptoms in childhood and adolescence (Birmaher et al., 1996) is added the extreme variability of symptoms in migrant youths (American Psychiatric Association, 2013, p. 167; Moro, 2009). For this reason, giving a full list of including and excluding symptoms is not possible. To improve the generalizability of our results, the depression diagnosis lays on gathering symptoms with a clinical global impression and an evaluation by the parents, but, to improve homogeneity, a nonexhaustive symptoms list is provided in Table 1 to help to do the diagnosis. Included participants must be aged 6–20 years old, be a first‐ or second‐generation migrant (born abroad or born from at least one parent who is born abroad), have a psychological and/or psychiatric follow‐up by a first‐line care, present a depression according the first‐line therapist based on usual as well as cultural symptoms criteria (Table 1), be rated at least “moderately ill” (score ≥ 4) on the severity scale of the improved Clinical Global Impression (iCGI) at inclusion, present transcultural issues confirmed by the referent (usual procedure of indirect preselection based on the presentation of the situation by referring physician), and have an informed consent signed by a legal tutor. Participants who have the following criteria cannot be included: patient or family who has previously had TCP, presents a severe mental disorder (e.g., excited delirium with great psychic disorganization and high suicidal risk) or somatic disease which may hinder the organization of the therapy, addressed for a legal expertize, refuses to participate.

**TABLE 1:** List of potential depressive symptoms

Sadness
Diminish interest or pleasure in most of the usual activities
Insomnia or hypersomnia
Psychomotor agitation
Asthenia, loss of energy
Feeling of worthlessness or excessive guilt
Recurrent thoughts of death
School problems (school failure, drop in grades, aggressivity with adult, school refusal…)
Mutism and selective mutism
Runaways
Aggressivity
Impulsivity
Violence and delinquency
Conflicts with parents and adults from the community
Exclusion from family
Somatic pains
Massive separation anxiety
Regressive symptoms—loosing of an already acquired function such as speech, walk, stay alone for a sufficient time in relation to age, manage stress or anxiety for reasonable situations…
Denial of medical care for a chronic disease with no evident reasons
Cultural designation such as possessed by a spirit, being a child witch, or other cultural designations
State of trance

#### 2.1.2 The first‐line therapist

Children and adolescents are referred to TCP consultations by a first‐line therapist who can work in a public hospital, a psychiatric and psychological center, in private practice, in a social or educational medical center. He is involved in individual semi‐structured interviews.

#### 2.1.3 Therapists of the transcultural group

All the therapists involved in TCP sessions will be involved in focus group interviews.

### 2.2 Treatment intervention, the TCP setting

TCP is a second‐line intervention. Physicians, psychologists, social worker, or child protection services refer patients to the transcultural consultation when they experience difficulties in building up a trustful relationship and good communication, when they feel helpless when confronted with cultural specificities of illness representation, or when they doubt about the pertinence of their diagnosis and the patient compliance with treatment and services. The evaluation of the referrals plays an important role. It may sometimes lead to an indirect consultation to help the professional who refer the patient to work in a more adequate way in their own consultation. When the TCP is proposed, the follow‐up should be provided by the initial care team who is invited to participate in the evaluation and to take part in the TCP.

This psychotherapy is composed of a 1‐hour session every 7 weeks. In these sessions, the patient and its family encounter a group of therapists of different cultural and linguistic backgrounds (psychiatry, psychology, anthropology, sociology, history, linguistics, or other scientific disciplines). The core family is invited with any member of the extended family or other people they feel close to. The group is a variable‐geometry setting which may vary from 2 to 10 participants, depending on the patient origin and the complexity of the situation. For example, a specific smaller group is designed to receive unaccompanied minors who could be intimidated with too many therapists. On the contrary, a bigger group is designed for receiving most of Sub‐Saharan African migrant families, as sickness is considered as a family and social concern and treated in the social group in many traditional societies. More information is provided in Appendix S1.

### 2.3 Randomization

Participants are randomized with a 1:1 allocation ratio to “usual care and waiting list for TCP for at least 34 weeks” (control group) or “usual care and TCP beginning in the 6 following weeks” (experimental group). Every participant continues his/her usual treatment with the first‐line medical team throughout the duration of the study. As it is a pragmatic real‐life study, usual treatment may vary from one patient to another and from one first‐line medical team to another. It may include psychotherapy (individual, in group, or family therapy), medications, or institutional care (day‐hospital or inpatient care).

The randomization is centralized, stratified according to the recruitment center, with an unknown block size except from the statistician of the research center unit who generated this allocation sequence independently from the investigators and the statistician of the study. The allocation sequence was prepared using NQuery Advisor^®^ v7.0, validated software using pseudorandom numbers generator, and the randomization is performed electronically (IWRS) using the Cleanweb application (Télémédecine) software.

### 2.4 Outcomes

#### 2.4.1 Primary endpoint

The severity score on the iCGI is used to determine the remission of the depression (Kadouri, Corruble, & Falissard, 2007). The iCGI is an improved version of the Clinical Global Impression (CGI) scale frequently used in medical care and clinical research because of its face validity and practicability. The CGI scale has notably shown its interest in differentiating responders versus nonresponders in clinical trials in depression (Guelfi, 1990). The iCGI has been constructed to improve its content validity and reliability in depressive disorders by the use of a filmed semi‐structured interview and a blinded scoring with a new response format (Kadouri et al., 2007). It has been used in different diseases, with children and adolescents or in cross‐cultural comparisons (Bailey et al., 2015; Bourredjem et al., 2011; Marquer et al., 2015).

The iCGI was chosen as many specific scales that exist to measure childhood depression have shown their limit in transcultural contexts (Bhugra et al., 2014; Lu, Lindsey, Irsheid, & Nebbitt, 2017; Marquer et al., 2015). Moreover, depressive disorders in migrant children may be complex with cultural issues and many comorbidities. In this context, iCGI has two advantages as follows: it is not specific to a pathology or a cultural context and is adapted for the study as it lays on a filmed semi‐structured interview, which has already shown its benefit in the diagnosis of depressive disorders in children and adolescents with migration backgrounds (Ceri et al., 2017).

The primary endpoint is measured at 28 weeks after the start of TCP for the experimental group, before the fifth therapy session, which corresponds to the 34th week (W34) after inclusion as a 6‐week delay is needed to organize the first TCP session. In the control group, it is measured 34 weeks after inclusion.

This measure consists in a filmed semi‐structured clinical interview conducted by a psychologist trained in transcultural care, in the presence of an interpreter speaking the native language of the family. The interview guide has been constructed by specialists of transcultural care in childhood and adolescence (Appendix S2). It explores the severity of the symptoms in the past few weeks with the patient and the family. After the end of the follow‐up of the last included participant, three blinded independent experts will watch the film and rate the patient on a 7‐level scale: 1—normal, not at all ill; 2—borderline mentally ill; 3—mildly ill; 4—moderately ill; 5—markedly ill; 6—severely ill; and 7—among the most extremely ill patients. Remission is defined as a mean iCGI score over the 3 experts lower than 4.

#### 2.4.2 Secondary endpoints

At each collection time points, the iCGI severity scale is used to measure the severity of depression, the Children's Depression Rating Scale‐Revised (CDRS‐R) is used to assess the level of depressive symptoms, and the State‐Trait Anxiety Inventory for Children (STAI‐C) is used to assess the level of anxiety symptoms (Spielberger, 1973).

The CDRS‐R is one of the most widely used rating scale for assessing severity of depression and change in depressive symptoms for clinical trials in children and adolescents (Mayes, Bernstein, Haley, Kennard, & Emslie, 2010). It's a 17‐item scale with answers on a 5‐ or 7‐point Likert scale depending on the item (total score ranges from 17 to 113). A score ≥40 indicates depression, whereas a score ≤28 is often used to define remission. This scale has shown good internal consistency and reliability for children over 6 years old (Poznanski & Mokros, 1996) as well as for adolescents (Mayes et al., 2010).

The STAI‐C is a self‐reported measure which has been widely used to assess state and trait anxiety of children and adolescents. It contains two scales of 20 items each, each subscale ranging from 20 to 60. The STAIC‐State scale is constructed to ask children how they feel at a particular moment in time. The STAIC‐Trait scale asks how they generally feel. Validity and reliability has been tested in many studies with children over 8 years old, adolescents and adults (Kirisci, Clark, & Moss, 1997).

Concerning the experimental group, a qualitative design is used to explore the therapeutic processes and the perceived efficacity and acceptability of the therapy. Sessions are transcribed verbatim and three semi‐structured interviews conducted by a psychologist after the fifth therapy session are organized with the family, the first‐line therapist and the transcultural group. The interviews' guides have been constructed by specialists of transcultural care in childhood and adolescence (Appendix S3).

### 2.5 Study schedule

#### 2.5.1 Screening time (indirect assessment without the patient)

The potential participants are referred to the different inclusion centers by a structured mail. A clinician evaluates the demand, the compatibility of the situation with the study, and contacts the family to propose an appointment with the research team in the next 5 weeks (Figure 1 and Table 2).

**TABLE 2:** Assessment prevision for each research meeting

	Screening W −5 to −1	Baseline W0	Visit 1 W6 (±7 days)	Visit 2 W13 (±7 days)	Visit 3 W20 (±7 days)	Visit 4 W27 (±7 days)	Visit 5 W34 (±7 days)	Visit 6 W35–44* (±7 days)	Visit 7 W52* (±7 days)
Location of the meeting	‐	I	I or F or H	I or F or H	I or F or H	I or F or H	I	I or F or H	I
Information	✓	✓	‐	‐	‐	‐	‐	‐	‐
Written consent	‐	✓	‐	‐	‐	‐	‐	‐	‐
iCGI assessment	‐	✓	✓	✓	✓	✓	✓	‐	✓
CDRS‐R assessment	‐	✓	✓	✓	✓	✓	✓	‐	✓
STAI‐C assessment	‐	✓	✓	✓	✓	✓	✓	‐	✓
Qualitative interviews*	‐	‐	‐	‐	‐	‐	‐	✓	‐
Transcultural psychotherapy session*	‐	‐	✓	✓	✓	✓	✓	‐	‐

Abbreviations: CDRS‐R, Children's Depression Rating Scale‐Revised; F, first‐therapy center; H, family's home; I, inclusion center; iCGI, improved Clinical Global Impression; STAI‐C, State‐Trait Anxiety Inventory for Children; W, week.

*In the experimental group only.

#### 2.5.2 Baseline visit and inclusion (week 0; duration: 1 h 30 min)

All the family members are invited to come in the recruitment center to meet a psychologist accompanied by an interpreter trained in transcultural care and briefed about the study. After information and general data gathering—questionnaire—the written consents (patient and family members, available in French on request) are collected, and the psychologist conducts a 5‐minute filmed semi‐structured interview with the participant and his/her family for the iCGI assessment.

For each participant fulfilling the inclusion criteria, randomization is then conducted. For each referred patient not included in the study, sociodemographic data and the reason for nonenrollment are collected.

#### 2.5.3 Weeks 6–34—Intervention follow‐up visits (0 h 30 min)

Participants from both groups continue their usual care provided by the referent medical team outside the inclusion center.

In the experimental group, participants receive TCP in addition to usual care, which consists of five sessions every 7 weeks (W6, W13, W20, W27, and W34) ± 1 week. The verbatim of all therapy sessions is collected depending of each group habits: audio recording and full transcription or verbatim transcription during the therapy session, as well as details of each session (number of therapists and their profession).

In both groups, an assessment is conducted by a psychologist at W6, W13, W20, W27, and W34 (before the therapy session in experimental group). The same assessment process as at the baseline visit is performed.

#### 2.5.4 Weeks 35–52—Free follow‐up

In the control group, TCP can be initiated or not, depending on clinical criteria. No new assessment will be performed after the W34 assessment. The research ends at W34 after the last measures for this group.

In the experimental group, TCP can be interrupted or continued depending on clinical criteria. Three qualitative semi‐structured interviews are organized between W34 and W44: one with the family (family interview); one with the therapists of the transcultural group (focus group); and one with the first‐line therapist(s) in charge of the participant who accompanied him during the therapy (individual or group interview depending on the case). These interviews explore the subjective experience of transcultural care, its acceptability, and the factors that each participant is highlighting as therapeutic in the TCP. The interviews are between 1 and 2 h long. At W52, a new assessment time is organized exactly in the same way as the previous follow‐up visits. The research ends at W52 after the last measures for this group.

### 2.6 Sample size

Eighty participants will be enrolled in the study: 40 in experimental group and 40 in control group.

In principle, the sample size in Bayesian designs do not need to be formally justified. Nonetheless, for information and for equivalence with a frequentist design, the following calculation was performed. An a priori distribution was estimated from the elicitation of expert opinions in a pilot study. To limit allegiance bias (associated with the experts' theoretical or treatment preferences), experts were asked to estimate the effect of the care as pessimistically as possible. The following distribution was found for estimates of the difference in the percentage of remission in the two randomized groups: 10%–0%: 0; 0%–10%: 1 expert; 10%–20%: 12 experts; 20%–30%: 4 experts; 30%–40%: 3 experts; 40%–50%: 0. The mean (±SD) of this distribution is 0.195 (±0.0825) and corresponds an a priori of 23 “virtual” subjects per group (the standard deviation 0.0825 being equal to sqrt(*p*[1−*p*]/*n*) where *p* = 0.195 and *n* is the number of virtual subjects per group; Spiegelhalter, Abrams, & Myles, 2004). Added to the 40 “real” subjects (included in this study), the a posteriori overall sample size will therefore be 63 subjects per group. In a frequentist design, a sample size of 63 per group would be sufficient to show a significant 23% difference in efficacy between groups (power = 80%, *α* = 0.05). As TCP is already used for a long time in practice in several centers, the Bayesian design is preferable for ethical reasons (less patients to include), for economical ones and for feasibility (centers could be reluctant to include too many patients).

### 2.7 Data management

In each center, the investigator or a collaborator will collect the data based on patient's medical file, interrogation on the cultural context and history of migration and questionnaires filled at each collection time (Appendix S4). All the data will be entered electronically via a web browser recorded in a e‐CRF (Cleanweb, Télémédecine). The access to the final trial dataset will be restricted to all the persons authorized by the sponsor in agreement with the scientific committee. This research is governed by the CNIL “Reference Method for processing personal data for clinical studies” (MR‐001). There will be no data monitoring committee.

### 2.8 Specific committees for the study

A steering committee and a scientific committee will be composed of at least the coordinating investigator, the methodologist, the scientific director (for the scientific committee), and the sponsor's appointed representatives for the study. They will meet once a year to define the overall organization of the study, coordinate the information, monitor the research process, suggest procedures to be followed during the study, recommend changes to the protocol during the study if necessary. Every important protocol modification will be amended on clinicaltrial.gov and submitted to CPP for approval.

No particular risk of any adverse event has been identified that could be caused by a psychotherapy properly conducted, however adverse events will be collected and described in the study report and any serious adverse event will be reported to the sponsor regardless of causality.

## 3 RESULTS

### 3.1 Quantitative analysis

Analyses will be performed according to the intention to treat (ITT) principle and no interim analysis will be performed. For the primary objective, as the iCGI severity score ≥4 is an inclusion criterion, all patients will have at least a measure at W0 and will be included in the ITT analysis. For patients with missing data on the iCGI assessment at W34, in case of less than 5% of missing data, the last observation carried forward will be used as imputation method, multiple imputations will be used otherwise (Jakobsen, Gluud, Wetterslev, & Winkel, 2017). For the secondary objectives, as mixed models will be used, missing data will not be imputed. For every objective, per protocol analyses will be performed as sensitivity analyses. The per protocol population is defined as patients for who the baseline and the last planned measure (W34) of the considered endpoint are available and who received the allocated intervention assigned by the randomization.

#### 3.1.1 Primary objective

A Bayesian design following the work of Spiegelhalter et al. (2004) is proposed to analyze the primary outcome. An a priori distribution for the difference in this percentage of patients in remission after 6 months between both groups has been estimated from the elicitation of 15 clinician's opinions. A structured questionnaire similar to the one used in Parmar, Spiegelhalter, Freedman, and Chart Steering Committee (1994) was used and a short video explaining the study, the Bayesian design and why a priori opinions of clinicians are required were send to the clinicians along with the questionnaires. According to the literature (Spiegelhalter et al., 2004, p. 141), clinicians were as numerous as possible. They were child and adolescent psychiatrists with at least five patients corresponding to the patients included in this study. This group was a mixture of clinicians who had never and who had already referred a patient to TCP. Voluntarily, these clinicians were not experts in TCP to reduce allegiance bias as possible, that is, they have not published on the topic, they were not specialized in TCP, and have not participated regularly (i.e., at least 1 or 2 weeks) to TCP sessions. They were recruited among first‐line care teams who have already referred patients to TCP. A pessimistic a priori distribution was obtained from the experimental a priori distribution, dividing the mean of efficacy by 2 (keeping the same variance).

The final decision concerning treatment effect will be made from the observation of the posterior distribution of the log odds ratio (mixture of two normal distributions weighted by the variances: observed data and a priori distribution), the pessimistic posterior distribution (mixture of observed data and pessimistic a priori distribution), and from the distribution of observed data (uninformative prior). The 95% credible interval of efficacy will be estimated in these three situations.

#### 3.1.2 Secondary objectives

They will be analyzed using a frequentist perspective, but they will be considered as exploratory in nature. For this reason, no adjustment for multiple testing will be done. Score (iCGI, CDRS‐R, and STAIC‐State) change from baseline to W34 will be analyzed using a mixed‐effects model for repeated measures with treatment group, visit, and treatment group‐by‐visit interaction as fixed effects and the baseline score and center as covariates. The persistence of the efficacy of TCP over time will be evaluated using paired Student's *t*‐test comparing the mean of the score (iCGI, CDRS‐R, and STAI‐C‐State) at W34 and W52 in the experimental group.

### 3.2 Qualitative analysis

The qualitative analysis will follow the interpretative phenomenological analysis (Smith, 2008) to study the meaning that the subject constructs from his or her own experiences. The transcribed verbatim of the interviews will be analyzed by three different researchers to optimize validity. This analysis will use the NVIVO^©^ software. Next, the textual data will be analyzed using the statistical clustering procedures encoded in ALCESTE^®^ software (Image, 2018). It constructs an overview of patterns found in the text (classes) according to how often roots and word forms appear together, for example, by performing different types of hierarchical descending classifications. Finally, the results of each analysis will be compared, and the quantitative results will be used to refining the qualitatively generated categories. Data from the therapeutic sessions as well as from each type of interview (family, first‐line therapist, or therapeutic group) will be pooled and compared during the analysis time.

### 3.3 Data integration

We will use a convergent and merged design with an integrated discussion. The quantitative and qualitative components will be analyzed separately. Their findings will be then compared to assess the complementarity, convergence, and divergence of the data. We will present the results of each component separately and then an integrated discussion.

## 4 DISCUSSION

In total, results will encourage the development and routinization of TCP for second‐line use and its adaption as a first‐line technique in this population. Today, the standard psychotherapeutic care offered for depression in children and adolescents does not lead to complete remission in patients with migration backgrounds, who present complex clinical depression with numerous comorbidities and requiring long care and substantial human and financial resources. This results in longer states of depression, which often become chronic. Morbidity is greater in the short, medium, and long term. In the short term, the risk of suicidal action rises, as do anxiety symptoms, repercussions on schooling (failures), and on social bonds (marginalization and violence). The consequences in adulthood on individual and social functioning and healthcare costs are large (Weissman et al., 1999). Every important trial result will be communicated to participants, healthcare professionals, and the public through publications, poster and oral communication in congress, and other media.

Benefits of the research for the patients will be faster improvement of depressive symptoms, directly affecting short‐ and long‐term morbidity. The benefits in terms of public health will also be important. The flow of migrant families and unaccompanied minors into France continues, coming from ever more distant geographic and cultural origins and often been exposed to complex traumatic situations. The numbers of descendants of migrants are increasing rapidly. The care offered to these children when they have resistant complex depression must include a cultural component (Georgiades, Paksarian, Rudolph, & Merikangas, 2018) to reduce both direct and indirect healthcare costs. The qualitative aspects will allow us to specify the therapeutic processes within this psychotherapy and make them more reproducible and transmissible: What are the principal therapeutic aspects in the visit? How can we extract them to make them more effective? Finally, the analysis of data about the acceptability of this psychotherapy will allow us to define the limits of its indications.

The heterogeneity of the population included (first‐ or second‐generation migrants, refugees, unaccompanied minors, etc.) is representative of the multiple situations usually encountered in the target population of TCP. Subgroup analyses will unfortunately not be sufficiently powered due to the small sample size. The diagnosis of depression will be made by the first‐line therapist referring the patient as diagnostic criteria for adolescent depression are too instable and are considerably influenced by culture. Clinical characteristics will be collected to describe the clinical pictures of the participants. Finally, the a priori distribution is obtained from expert's opinion and not from any source of objective data. This can be considered obviously as a methodological limitation, but it is also a way to include all source of knowledge in the evaluation of a treatment, which is ethically interesting.

## CONFLICT OF INTERESTS

All authors have completed the ICMJE uniform disclosure form at www.icmje.org/coi_disclosure.pdf and declare no support from any organization for the submitted work; no financial relationships with any organization that might have an interest in the submitted work in the previous 3 years; no other relationships or activities that could appear to have influenced the submitted work.

## Supporting information

Appendix S1 The transcultural psychotherapy setting

Appendix S2 iCGI protocol

Appendix S3 Semi‐structured interview guides

Appendix S4 Questionnaire of sociodemographic characteristics and clinical history of the depression
