# Supplementary material for: Assessment of transcultural psychotherapy to treat resistant major depressive disorder in children and adolescents from migrant families: Protocol for a randomized controlled trial using mixed method and Bayesian approaches
Source: Int J Methods Psychiatr Res. 2020 Sep 12;29(4):e1847. doi: 10.1002/mpr.1847 (PMC7723212; doi:10.1002/mpr.1847)
Supplement: Supplementary file 1 — Appendix S1 The transcultural psychotherapy setting [file MPR-29-e1847-s001.docx]

**Appendix 1 – The transcultural psychotherapy setting**

One of the therapists is called the main therapist, leads the encounter and distributes the floor. Co-therapists provide him/her comments, representations, symbolizations, metaphors, or interpretations of any aspects of the patients’ discourse. All the words are directed to the main therapist so that he/she has the power to reinforce or soften any proposal before giving it back to the patient. This rule is essential to protect the patients from aggressive, insensitive or chaotic interventions (for more details, refer to published manual of TCP (Moro, 2020)).

The group has four fundamental functions (Moro, 1998). At a general level, it has a function of holding and psychic surrounding, which allows communication and elaboration for the parent, who often suffers from trauma, and for the child recognized in its otherness (Anzieu, 1995; Winnicott, 1957). At a cultural level, as the group is the way of hearing and treating illness in traditional societies, it may be more comfortable for migrant persons to deliver their intimacy to a group than to a single therapist (Thomas & Luneau, 1975). Third, the multicultural group helps the decentring process in materializing otherness – i.e. group of therapists from different backgrounds with different origins, speaking different languages… –. Lastly, the group proposes multiple different – and sometimes conflicting – ways of thinking illness. This process allows to access to the insight of patients, their psychic conflicts and complex representation of the world.

The main therapeutic work lays on dialoguing between cultural meanings of the illness, traditional etiologies of suffering and western way of considering the medical care. The content of the consultations are narratives of the families’ history, of the migration, and of the confrontation to European way of living and understanding of the world. The two targets of the therapy are traumatic aspects of the migration and psychic cleavage.

Another main specificity of the transcultural setting is the systematic use of interpreters speaking in the family’s mother-tongue. Depending on the family’s fluency in French, the interpreter may translate all the consultation word-to-word, or they may be solicited for some specific traumatic, emotional or intimate sentences. Research studies on working with interpreters in child mental health have shown that interpreters have a great role in therapeutic alliance (Leanza et al., 2015; Rousseau, Measham, & Moro, 2011). They are a key figure for children as someone who can help to understand their parents’ representations and who make links between the inner world and the outside world. They are solicited as cultural informants and might be considered as co-therapists.

Anzieu, D. (1995). *Le Moi-peau* (Édition : 2e). Paris: Dunod.

Leanza, Y., Boivin, I., Moro, M. R., Rousseau, C., Brisset, C., Rosenberg, E., & Hassan, G. (2015). Integration of interpreters in mental health interventions with children and adolescents : The need for a framework. *Transcultural Psychiatry*, *52*(3), 353‑375. doi: 10.1177/1363461514558137

Moro, M. R. (1998). *Transcultural psychotherapy of migrants children [Psychothérapie transculturelle des enfants de migrants]*. Dunod.

Moro, M. R. (2020). *Guide de psychothérapie transculturelle : Soigner les enfants*. Paris: In Press.

Rousseau, C., Measham, T., & Moro, M. R. (2011). Working with Interpreters in Child Mental Health : Interpreters in Child Mental Health. *Child and Adolescent Mental Health*, *16*(1), 55‑59. doi: 10.1111/j.1475-3588.2010.00589.x

Thomas, L.-V., & Luneau, R. (1975). *La terre africaine et ses religions*. Paris: Editions L’Harmattan.

Winnicott, D. W. (1957). *The child and the family : First relationships*. Tavistock Publications.
