# Supplementary material for: Assessment of transcultural psychotherapy to treat resistant major depressive disorder in children and adolescents from migrant families: Protocol for a randomized controlled trial using mixed method and Bayesian approaches
Source: Int J Methods Psychiatr Res. 2020 Sep 12;29(4):e1847. doi: 10.1002/mpr.1847 (PMC7723212; doi:10.1002/mpr.1847)
Supplement: Supplementary file 2 — Appendix S2 iCGI protocol [file MPR-29-e1847-s002.docx]

**Appendix 1 – iCGI-Severity scale and semi-structured interview**

**Semi-structured interview guide for iCGI cotation**

The clinical interview has been adapted from Kadouri et al (2007) depressive disorders interview. It is a semi-structured interview close to day-to-day clinical practice, and it is divided into three stages. The interview guide has been constructed by specialists of transcultural care in childhood and adolescence.

The interview is held with the child or adolescent and their families, excepted for non-accompanied young people whom are interviewed alone. The interviewer addresses the young people or the parents depending on the age and on the children’s easiness. Questions are adapted to the interlocutor.

*First question*

Addressing the young people: "Can you tell me how you are, and what is going well and what isn't just now?"

Addressing the parents: "Can you tell me how your son/daughter is, and what is going well and what isn't for him/her just now?"

*The second stage* corresponds to the main body of the interview. The objective is not to direct the participants towards particular areas but rather to make the interview as sensitive as possible. The interviews explore the main issues concerning the young people and the family, and what changed and what didn’t change since the inclusion.

During the baseline visit, the main symptoms presented by the participant are gathered, on the basis of a symptom list – presented in table 1 –. During this stage of the interview, the researcher asks questions with the objective to evaluate the intensity of these symptoms and their evolution since the last research visit. For example, if one of the main symptoms is school problems, the researcher would ask some news about the school, if the child went to school regularly or did not, if the if there were not any behavioral symptoms with professors… If one of the main symptoms is recurrent states of trance, the interviewer may ask for frequency, duration of the states, intensity of behavioral symptoms…

*The third stage* is the end of the clinical interview. The final question can be "Can

you say something else about your (son/daughters’) situation?"

**Film procedure and digitalization**

The patient and the family are filmed during the interview. The film is cut during digitization. The family is filmed three-quarters turned or full-face, in static shot, with chest, head and hands in the same frame at each visit so that non-verbal behaviours can be captured. Any information which might reveal the visit number is deleted from the final videos.

The first 5 min of the video are kept for rating. Information related to the treatment group is deleted from the final video. Videos are copied to a DVD.

**Scoring**

3 independent experts blinded to patients’ treatment status will watch the film and rate the patient using the following improved (compared to CGI) response format: 1-Normal, not at all ill, 2-Borderline mentally ill, 3-Mildly ill, 4-Moderately ill, 5-Markedly ill, 6-Severely ill, 7-Among the most extremely ill patients. The average of the three scores is considered as the final measurement.
