# Supplementary material for: Assessment of transcultural psychotherapy to treat resistant major depressive disorder in children and adolescents from migrant families: Protocol for a randomized controlled trial using mixed method and Bayesian approaches
Source: Int J Methods Psychiatr Res. 2020 Sep 12;29(4):e1847. doi: 10.1002/mpr.1847 (PMC7723212; doi:10.1002/mpr.1847)
Supplement: Supplementary file 3 — Appendix S3 Semi‐structured interview guides [file MPR-29-e1847-s003.docx]

**Appendix 2 – Qualitative semi-structured interviews**

**Semi-structured interview guide with families**

- How have you been told about the transcultural consultation and what helped you to decide to accept the therapy?

- Can you tell us how was the first session? What did you feel during the consultation?

- What did the transcultural psychotherapy do on your child? On the family? What changes occurred during the follow-up period?

- In your opinion, which issues of the therapy are involved in these changes?

- What problems did you encounter during the follow-up?

- What improvements could be made to the transcultural therapy?

**Semi-structured interview guide with first-line medical team**

- What clinical and other elements enabled you to refer your patient to the transcultural consultation?

- Can you tell us how was the first session? What did you feel during the consultation?

- What did the transcultural psychotherapy do on your patient? On the family? On the therapeutic relationship? What changes occurred during the follow-up period?

- In your opinion, which issues of the therapy are involved in these changes?

- What problems did you/your patient encounter during the follow-up?

- What improvements could be made to the transcultural therapy?

**Semi-structured interview guide with group of transcultural psychotherapists**

- What clinical and other elements enabled the first line therapists to refer the patient to the transcultural consultation?

- Can you tell us how was the first session? What did you feel during the consultation?

- What did the transcultural psychotherapy do on your patient? On the family? On the therapeutic relationship? What changes occurred during the follow-up period?

- In your opinion, which issues of the therapy are involved in these changes?

- What problems did you/your patient encounter during the follow-up?

- How may we improve the care for this family?
