# Supplementary material for: Assessment of transcultural psychotherapy to treat resistant major depressive disorder in children and adolescents from migrant families: Protocol for a randomized controlled trial using mixed method and Bayesian approaches
Source: Int J Methods Psychiatr Res. 2020 Sep 12;29(4):e1847. doi: 10.1002/mpr.1847 (PMC7723212; doi:10.1002/mpr.1847)
Supplement: Supplementary file 4 — Appendix S4 Questionnaire of sociodemographic characteristics and clinical history of the depression [file MPR-29-e1847-s004.docx]

**Appendix 3 – Questionnaire of socio-demographic characteristics and clinical history of the depression**

**I Socio-demographic data**

1. Gender
2. Age
3. Brothers and sisters and their age
4. Family situation: Parents are Married/PACS/Common-law, Separated, Lone parent…
5. **If non-accompanied minor or young adult**: Marital status: Married/PACS/Common-law, Separated, Single, …
6. **If non-accompanied minor or young adult**: Social Status ( Minor supported by French social services (Aide Sociale à l’enfance, ASE); Adult helped by ASE (contrat jeune majeur); Adult without social accompaniment)
7. Academic level of the parent(s)
8. Home: The child/adolescent lives with who
9. School level (grade) or professional status
10. **If working**, academic level
11. Grade repetition? If yes, which grade
12. Out-of-school / De-schooling? If yes when, how long and why?
13. Leisure outside the house, school/college/high school? If yes, which one(s)?
14. Mother tongue of the adolescent
15. What language(s) is(are) spoken at home?

NB: **If non-accompanied minor or young adult**: Languages spoken fluently since childhood

1. The Child/adolescent is:

- Born in France from two parents born abroad

- Born in France from one parent born in France and one parent born abroad

- Born abroad (if yes, number of years in France)

**II Symptoms**

1. Family history of mental disorders
2. Personal history of mental disorders
3. Duration since the apparition of the first symptom
4. Symptoms description: Presence or absence of

- Sadness
- Diminish interest or pleasure in most of the usual activities
- Insomnia or hypersomnia
- Psychomotor agitation
- Asthenia, loss of energy
- Feeling of worthlessness or excessive guilt
- Recurrent throughs of death
- School problems (school failure, drop in grades, aggressivity with adult, school refusal…)
- Mutism, and selective mutism
- Runaways
- Aggressivity
- Impulsivity
- Violence and delinquency
- Conflicts with parents and adults from the community
- Exclusion from family
- Somatic pains
- Massive separation anxiety
- Regressive symptoms – loosing of an already acquired function such as speech, walk, stay alone for a sufficient time in relation to age, manage stress or anxiety for reasonable situations…
- Denial of medical care for a chronic disease with no evident reasons
- Cultural designation such as possessed by a spirit, being a child witch, or other cultural designations
- State of trance
- Other : ……

**III Medical care**

1. First contact with a psychiatrist/psychologist
2. Number of previous psychic care programs
3. Duration of current care
4. Type(s) of follow-up(s) related to the symptoms (psychiatric, psychological…)
5. Drug treatment
6. Inpatient treatment
7. Location(s) of the current care (outpatient care or inpatient care)

**IV Anthropological issues**

1. Rationale for Transcultural Therapy Consultation Orientation

- A traditional theory is advanced by the family to explain the child's disorders, and this theory conflicts with medical care

- The family follow a traditional treatment which prevent the successful conduct of medical care (the treatment conflicts with medical prescriptions, prevent a good alliance, …)

- Cultural misunderstandings are present and hinder the successful conduct of medical care

- The family conflicts with the medical team and the conflict seems to be based on cultural elements

- There are intra-family conflicts around cultural issues and the first-line team seems unable to fix them in the regular follow-up

- There is a traumatic migration experience that cannot be treated in individual follow-up and requires a group therapy

- ...
